# A warning against over-interpretation of seasonal signals measured by the Global Navigation Satellite System

**DOI:** 10.1038/s41467-020-15100-7

**Published:** 2020-03-13

**Authors:** Kristel Chanard, Marianne Métois, Paul Rebischung, Jean-Philippe Avouac

**Affiliations:** 1 Université de Paris, Institut de physique du globe de Paris, CNRS, IGN, F-75005 Paris, France; 2 Laboratoire de géologie de Lyon, Université de Lyon, Université Lyon 1, ENS de Lyon, CNRS, UMR 5276 LGL-TPE, F-69622 Villeurbanne, France; 3 Geological and Planetary Sciences, California Institute of Technology, Pasadena, CA USA

**Keywords:** Solid Earth sciences, Tectonics

**Arising from** D. Panda et al. *Nature Communications* 10.1038/s41467-018-06371-2

In a recent study, Panda et al.^1^ claim that seasonal strain across the Himalaya indicates seasonal slow slip on the Main Himalayan Thrust (MHT) fault driven by hydrological loading related to the monsoon and driving seasonal variations of seismicity. While we find the analysis interesting, we spell out some reasons why the claim should be considered with caution.

Global Navigation Satellite Systems (GNSS) station position time series exhibit strong seasonal horizontal and vertical signals^2^. These signals have been primarily attributed to annual surface mass redistribution of continental hydrology, ice and snow, non-tidal and atmospheric pressure^3^. A number of studies have shown that these signals can be modelled to first order as the response of a spherically layered elastic Earth to an integrated surface mass loading derived from the Gravity Recovery and Climate Experiment (GRACE)^4^ or a combination of hydrological, atmospheric and oceanic loading models^5^.

Panda et al.^1^ find the horizontal seasonal geodetic signal in the Garhwal-Kumaun and Nepal Himalaya to be significantly larger than predicted by such models. They assume that, in absence of seasonal slow slip on the MHT, and considering predictions from hydrological and atmospheric loading models, the ratio of the annual amplitude of horizontal over vertical displacements (H/V) should not exceed a value of 0.5.

First, it should be noted that current surface load models explain only up to 30% and 50% of the annual amplitudes of seasonal horizontal and vertical GNSS observations, respectively, at the global scale^2,4–6^. Much of the residual seasonal signals are likely caused by unmodelled geophysical signals, and/or GNSS errors rather than localized tectonic motion. We therefore find it useful to review the potential sources of short spatial wavelength (less than a few hundred km) or site-dependent signals that could affect the H/V seasonal ratio locally. Their non-negligible contribution results in H/V often exceeding 0.5 for a globally distributed network of stations, even in regions where no significant tectonic motion is expected (Fig. 1).

**Fig. 1:**
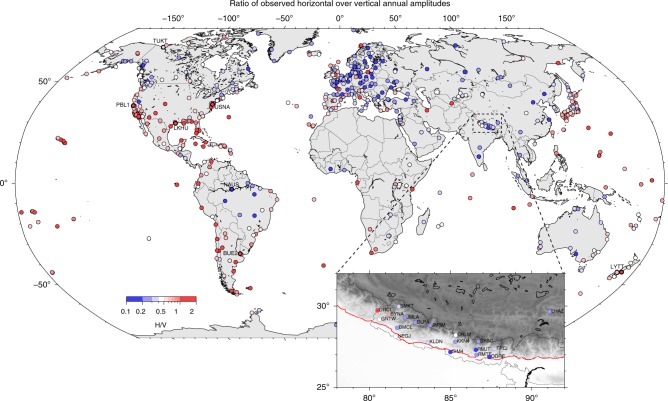
Observed horizontal over vertical annual amplitudes (H/V) ratio. H/V ratio is shown at a set of 689 globally distributed Global Navigation Satellite Systems (GNSS) stations of the International GNSS Service (IGS) that were a part of the second IGS reprocessing campaign (see Data Availability). Stations for which the North component seasonal signal is shown in Fig. 2c are indicated as bold dots and labelled with their site names. The same ratio is also shown for 18 GNSS stations located in Nepal. Ratios often exceed 0.5 at the global scale, in regions where not tectonic deformation is expected. In Nepal, two stations exhibit higher than 0.5 H/V ratio, in a limited geographic location.

Several geophysical sources, in addition to surface mass variations, can induce seasonal horizontal and vertical displacements at short spatial wavelengths. Seasonal variations in the Earth temperature field induce a thermoelastic deformation of GNSS monuments^7^ and of the bedrock^8^, both estimated to reach up to ~1 mm vertical amplitude. Moreover, thermoelastic deformation, using a realistic Earth model, can induce up to a few millimetres of horizontal displacements at short spatial wavelengths due to lateral heterogeneities of shallow mechanical properties of the Earth^9^. Similarly, poroelastic deformation may induce comparable displacements in areas with large variations of the water table^10^. Stations suggested to exhibit seasonal slow slip motion by Panda et al.^1^ in Nepal (BMCL and DRCL) are reported to be installed on sedimentary or weathered metamorphic bedrock (see Data Availability), possibly enhancing both thermoelastic and poroelastic seasonal deformation, and contributing to larger H/V ratios.

In addition to these geophysical sources, systematic errors in GNSS observations and in their modelling may induce station-dependent seasonal signals. Unmodelled or mis-modelled semi-diurnal and diurnal tides may for instance alias into millimetric annual vertical signals due to the beating with both the GNSS satellite ground repeat period and the processing of GNSS observations in 24-h batches^11^. Tropospheric delay mismodelling may also be responsible for millimetric annual vertical signals^12^. Environmental effects such as snow and ice cover, soil moisture or vegetation growth additionally influence the GNSS antenna phase centers and local multipath, and may result in apparent seasonal station displacements. Such station-specific effects are hard to quantify globally, but examples exist of environmental changes causing centimetric station position variations^2^. Besides, spectral analyses of GPS time series have revealed spurious periodic signals with millimetric amplitudes at harmonics of the GPS draconitic year (≈351.6 days), i.e. the period at which the orientation of the GPS constellation with respect to the Sun repeats^13^. Possible causes for those draconitic signals are orbit modelling deficiencies (e.g. solar radiation pressure or eclipse mismodelling) and the aliasing of station‐dependent errors (multipath, antenna phase centre mismodelling) through 24-h sampling. Draconitic errors can in principle be separated from seasonal variations in long enough time series. They may otherwise interfere with seasonal variations and bias their interpretation.

Second, there is no reason that, in absence of subsurface sources of deformation, the H/V ratio should not exceed the particular value of 0.5 chosen by Panda et al.^1^. While the H/V ratio would be constant for a surface point source loading a homogeneous elastic half-space Earth model (and equal to (1–2*ν*)/2(1–*ν*) ~0.33, for a Poisson coefficient *ν* of 0.25), an assumption that has been shown to perform poorly for modelling seasonal GNSS observations, it varies with the distance from the loads for a more realistic spherically layered Earth model. It peaks to 0.5 for a PREM layered structure and may exceed this value depending on the local depth variations of elastic properties^14^. Figure 2a, b is an attempt at reproducing Fig. 4 of Panda et al.^1^. Figure 2a shows that, once we added error bars on H/V ratios (see Fig. 2 caption for details), which were not included in the original figure of Panda et al.^1^, only two stations (DRCL and BYNA) have an H/V ratio significantly larger than 0.5. Figure 2b, c shows GNSS time series stacked over a year and two loading model results, respectively, for Nepal stations and a selection of GNSS sites around the globe in tectonically stable areas where the discrepancy between the observed and modelled seasonal signal is as large or larger than at DRCL. Affirming that the seasonal horizontal geodetic positions is due to seasonal slow slip motion because it cannot be predicted by a particular choice of hydrological and atmospheric models disregards the high variability and imperfect nature of existing surface loading models at the global scale, particularly where no tectonic deformation is expected (Fig. 2c).

**Fig. 2:**
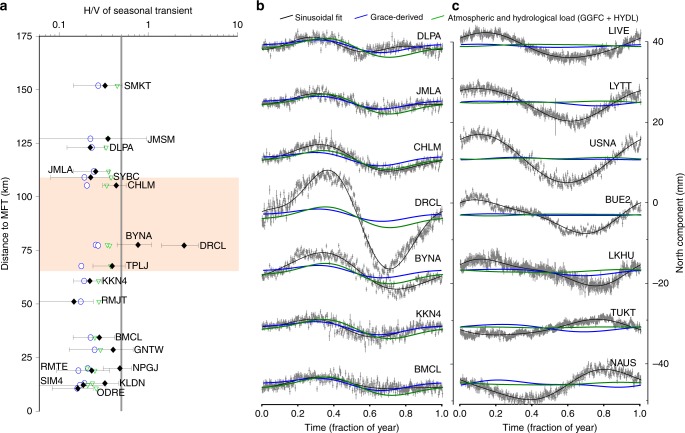
Measured annual signals and modelled surface loading contribution. **a** Ratios of mean amplitudes of horizontal over vertical annual signals (H/V ratio) with distance to the Main Frontal Thrust (MFT) for Global Navigation Satellite Systems (GNSS) observations (see Data Availability) in Nepal (black diamonds), Gravity and Recovery Climate Experiment (GRACE) derived loading model (blue contoured dots; Chanard et al.^4^) and the sum of HYDL (hydrological loading model) and GGFC (see Data Availability) (Global Geophysical Fluid Centre) atmospheric loading (green contoured triangles). Mean annual peak to peak seasonal amplitudes and associated error bars have been estimated using the coordinate time series analysis software, CATS (see Data Availability), and combined in H/V ratio. The amplitudes of annual and semi-annual sinusoidal signals are estimated together with white and flicker noise from detrended time-series. **b**, **c** GNSS time series stacked over a year (grey dots and associated error bars), with corresponding sinusoidal fit (plain black), GRACE-derived model (plain blue) and the sum of HYDL hydrological loading model and GGFC (see Data Availability) atmospheric loading (plain green), models for Nepal (**b**) and a set of globally distributed International GNSS Service (IGS) stations (see Fig. 1, IGS, repro2) (**c**).

Finally, we recall that the seasonal variation of seismicity is not peculiar to the area of western Nepal around DRCL and BYNA and can be explained as a direct effect of the stress variations induced by seasonal loading^15^ without requiring slow slip on the MHT.

In light of the difficulty in accounting fully for the seasonal signals present in GNSS time series, whether due to a true response to seasonal load variations or to technical artefacts, we suggest that Panda et al.'s^1^ claim for seasonal slow slip on the MHT should be treated with caution. This claim hinges on the particularly high seasonal amplitude and H/V ratios observed at two stations. Such anomalies certainly deserve scrutiny but are not unusual globally and probably not related to tectonics in general. While progress has been made towards a better understanding and modelling of GNSS seasonal signals since Dong et al.^2^, they remain only partially understood, particularly for the horizontal components, and should be further investigated.

## Data Availability

Stations logs are available at: https://www.unavco.org/data/gps-gnss/data-access-methods/dai1/recent.php. Daily IGS repro2 station positions available at: https://cddis.gfsc.nasa.gov/gnss/products/repro2. Global Navigation Satellite System (GNSS) time series available at: http://geodesy.unr.edu/index.php. Hydrological loading model (HYDL) available at: http://rz-vm115.gfz-potsdam.de:8080/repository. Global Geophysical Fluid Center (GGFC) atmospheric model available at: http://geophy.uni.lu/ggfc-atmosphere/ncep-loading.html. CATS: GPS coordinate time series analysis software available at: https://www.ngs.noaa.gov/gps-toolbox/cats.htm
